# Bridging E3 Ligase Binding and Targeted Degradation Through Fluorescence Polarization

**DOI:** 10.3390/ijms27188054

**Published:** 2026-09-10

**Authors:** Lucía González-Pico, Tamara Camino, Sandra Ortigueira, Rubén Prieto-Díaz, Eddy Sotelo, Maria Majellaro

**Affiliations:** 1 Center for Research in Biological Chemistry and Molecular Materials (CIQUS), University of Santiago de Compostela, 15782 Santiago de Compostela, Spain; 2 Department of Organic Chemistry, Faculty of Pharmacy, University of Santiago de Compostela, 15782 Santiago de Compostela, Spain; 3 Celtarys Research SL, Avenida de Barcelona 7, 15706 Santiago de Compostela, Spain

**Keywords:** targeted protein degradation (TPD), PROTACs, molecular glues, fluorescence polarization, E3 ligases, von Hippel–Lindau (VHL), cereblon (CRBN), binding assay

## Abstract

Despite the rapid expansion of targeted protein degradation (TPD), early characterization of PROTACs and molecular glues still relies predominantly on cellular degradation assays, making it difficult to determine whether poor degradation results from suboptimal E3 ligase engagement or from subsequent steps in the degradation mechanism. Moreover, productive degradation depends on balanced binary interactions, as both insufficient and excessively strong binding to either the E3 ligase or the protein of interest (POI) can impair degrader activity. Here, we report robust fluorescence polarization (FP) assays for the quantitative determination of ligand binding to the two most widely exploited E3 ligases, cereblon (CRBN) and von Hippel–Lindau (VHL). Using proprietary fluorescent tracers, the assays were validated with chemically diverse molecular glues, clinically relevant PROTACs, and canonical ligands, yielding affinity values in excellent agreement with published data. Both assays were successfully miniaturized to 384-well format while maintaining analytical performance and subsequently implemented in standardized ready-to-use formats, facilitating reproducible affinity measurements across laboratories. These assays establish a rapid and accessible binding-first platform that complements cellular degradation studies; supports rational degrader discovery, profiling, and optimization; and enables the identification of structurally novel E3 ligase recruiters.

## 1. Introduction

Targeted protein degradation (TPD) has emerged as one of the most transformative strategies in modern drug discovery. Based on the concepts of event-driven pharmacology and induced proximity, TPD promotes the selective elimination of a protein of interest (POI) by hijacking cellular systems responsible for protein degradation [1]. Among the different TPD modalities, degraders acting through the ubiquitin–proteasome system (UPS) have attracted the greatest attention from both academia and the pharmaceutical industry, culminating in the recent FDA approval of the estrogen receptor degrader vepdegestrant for the treatment of ER-positive, HER2-negative, ESR1-mutated advanced or metastatic breast cancer, providing the first clinical validation of this therapeutic strategy [2,3].

Two major classes of UPS-based degraders have emerged: proteolysis-targeting chimeras (PROTACs) and molecular glue degraders (MGDs). PROTACs are heterobifunctional molecules composed of a ligand for the POI, an E3 ligase ligand, and a linker connecting both pharmacophores. Their modular architecture enables the rational combination of potentially any target ligand with an E3 ligase recruiter, greatly expanding the scope of druggable proteins [4,5]. However, this architecture also introduces several design challenges. Successful degradation requires not only sufficient affinity for both the POI and the E3 ligase, but also the formation of a productive ternary complex with an appropriate geometry that enables ubiquitin transfer to a lysine residue available on the POI. Consequently, binary affinity alone does not guarantee degradation, while excessive binary complex formation may even reduce degradation through the well-known hook effect, particularly at high PROTAC concentrations [6]. By contrast, molecular glues are monovalent small molecules that initially bind only to an E3 ligase and subsequently remodel its surface to promote de novo protein–protein interactions with a substrate or neo-substrate [7]. As molecular glues lack intrinsic affinity for the POI, productive ternary complex formation depends almost entirely on the structural changes induced upon E3 ligase binding [8]. Accordingly, many molecular glues have historically been discovered either serendipitously or through systematic optimization of known molecular glue scaffolds, particularly immunomodulatory imide drugs (IMiDs) [9,10].

Compared with conventional inhibitors, degraders offer several therapeutic advantages, including activity at extremely low cellular concentrations thanks to their catalytic behavior, suppression of both enzymatic and scaffolding functions, prolonged pharmacodynamic responses, the possibility of overcoming resistance mutations, and enhanced selectivity arising from cooperative ternary complex formation [5]. As a result, the field has experienced remarkable expansion over the last decade, with hundreds of degraders reported and an increasing number progressing into clinical development. However, this rapid growth has also shifted medicinal chemistry priorities. Whereas the first generation of degraders largely relied on a limited collection of well-established E3 ligase ligands—primarily VHL and CRBN recruiters—and a relatively small number of validated exit vectors, current efforts increasingly focus on expanding the chemical space of E3 ligase recruiters, while exploring alternative exit vectors, innovative linker architectures, and previously unexplored E3 ligases [11].

This shift is also driven by the current predominance of CRBN-based degraders, largely attributable to the favorable physicochemical properties of CRBN ligands, which have enabled the development of most orally bioavailable PROTACs that have reached clinical trials [12]. In contrast, the larger and structurally more complex VHL ligands have made the development of orally bioavailable VHL-based degraders considerably more challenging. Nevertheless, the ongoing clinical evaluation of the VHL-recruiting BCL-XL degrader DT2216 demonstrates the therapeutic potential of this ligase while underscoring the need for improved VHL-binding scaffolds capable of expanding the pool of VHL-based PROTACs in clinical trials [13]. Therefore, the discovery and rapid profiling of novel E3 ligase-binding scaffolds have become increasingly important, not only to enable next-generation orally bioavailable VHL degraders, but also to expand the chemical diversity, intellectual property landscape, and therapeutic versatility of CRBN-recruiting degraders.

As proven by the clinical candidates, even subtle modifications of the E3 ligase ligand can profoundly influence degrader behavior. Alternative exit vectors on VHL ligands have been shown to alter degradation efficiency, selectivity and substrate preference of JQ1-derived PROTACs despite preserving the same POI warhead, demonstrating that optimization of E3 ligase engagement plays a key role in degrader performance [14,15]. UPS-based degraders induce the degradation of POIs by simultaneously engaging an E3 ligase and the target protein. PROTACs are heterobifunctional molecules composed of a ligand for the POI and an E3 ligase recruiter connected by a linker, whereas molecular glues are small molecules that stabilize or induce protein–protein interactions between an E3 ligase and a POI. Regardless of their modality, both classes rely on the optimization of two independent binding interfaces, making degrader design conceptually analogous to the medicinal chemistry principles traditionally applied to dual-target drugs [16]. In the latter, affinity toward each individual target is routinely characterized and optimized before cellular efficacy is evaluated, enabling systematic structure–activity relationship (SAR) studies and rational compound optimization [17]. Surprisingly, despite PROTACs also requiring productive interactions with two independent proteins, degrader discovery still relies predominantly on cellular degradation assays as the primary screening strategy. Western blotting, capillary electrophoresis, BRET and related methodologies have become the standard techniques for identifying active degraders, while quantitative evaluation of binary binding is frequently omitted [18]. This workflow presents several important limitations. Failure to induce degradation may originate from numerous independent factors, including insufficient cell permeability, poor metabolic stability, low or excessive affinity for either the POI or the E3 ligase, unproductive ternary-complex geometry, inefficient ubiquitination, or impaired proteasomal processing [19]. Cellular degradation assays alone cannot distinguish among these possibilities, making structure–activity relationship interpretation considerably more challenging and often leading to unnecessary synthesis and biological evaluation of compounds with suboptimal physicochemical or biophysical properties [18].

**Figure 1:**
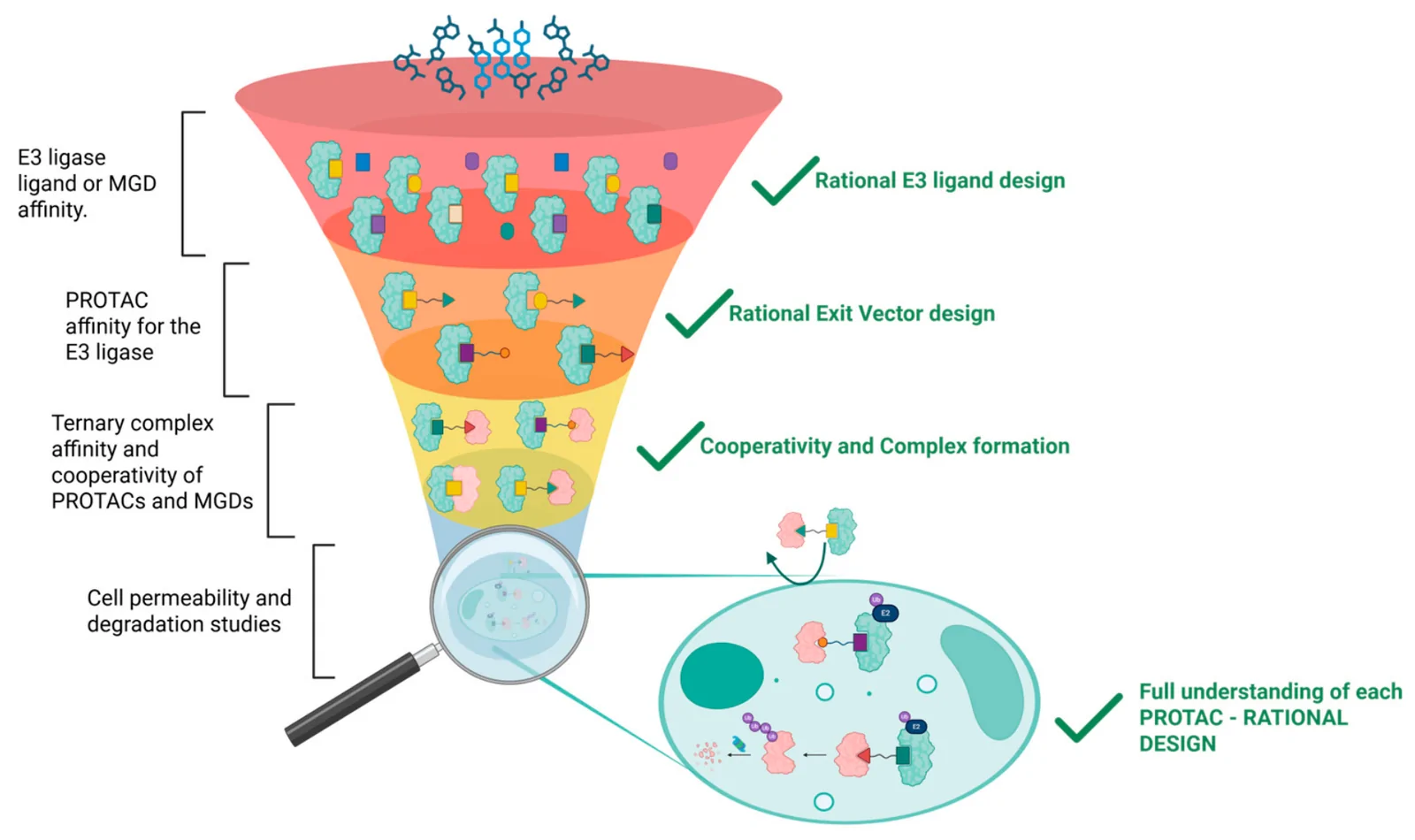
**Binding-first workflow for rational degrader discovery.** Conventional degrader discovery primarily relies on cellular degradation assays as the initial screening strategy (“degradation-first”), making it difficult to distinguish whether inactive compounds fail because of insufficient E3 ligase binding, poor target engagement, unfavorable ternary-complex formation, limited cell permeability, or other downstream factors. In the proposed “binding-first” workflow, quantitative fluorescence polarization (FP) assays are introduced as an early biophysical filter to characterize binary binding to the E3 ligase before cellular degradation studies. Early assessment of E3 ligase affinity enables rapid structure–activity relationship (SAR) development; supports the identification and optimization of novel E3 ligase recruiters, molecular glues, and PROTACs; and complements subsequent mechanistic studies, including ternary-complex formation, cooperativity, and cellular degradation.

Importantly, binary binding should not be regarded as a surrogate for degradation, but rather as the first experimentally accessible checkpoint along the degrader mechanism of action. Measuring E3 ligase affinity cannot always predict degradation efficacy by itself; however, it enables the identification of molecular liabilities that cannot be inferred from cellular degradation assays alone and therefore supports rational optimization of degrader architectures before progressing to more resource-intensive biological studies (Figure 1) [20]. The need for such rational screening strategies is becoming increasingly important as degrader discovery moves beyond simple “plug-and-play” designs. Early PROTAC development was largely based on combining established warheads with validated VHL or CRBN ligands using flexible linkers, allowing rapid proof-of-concept studies [21,22]. Today, however, medicinal chemistry efforts increasingly seek to explore novel E3 ligase ligands, unconventional exit vectors, differentiated linker chemistries and alternative degradation mechanisms. These innovations require rapid, quantitative and broadly accessible methodologies capable of guiding rational optimization at the earliest stages of drug discovery rather than relying exclusively on degradation outcomes [21]. In this context, recent systematic studies in which binary affinity, ternary-complex formation, cooperativity and degradation were evaluated in parallel have demonstrated that binary affinity contributes to degrader performance. Such is the case of Ichikawa et al.’s work, where cyclimids were assessed as CRBN recruiters, and a correlation between degradation activity and affinity for the E3 ligase was found [20]. Moreover, the relevance of binary affinity differs between degrader modalities. While PROTAC activity ultimately depends on the interplay between binary binding, ternary-complex formation, cooperativity, ubiquitination efficiency and proteasomal recognition, the majority of molecular glues initiate their mechanism through E3 ligase engagement before substrate recruitment can occur. Consequently, quantitative assessment of E3 ligase binding represents a critical early parameter for both degrader classes, although its mechanistic contribution differs between them [23,24].

Here, we describe robust fluorescence polarization (FP) binding assays for the quantitative determination of ligand binding to the two most widely exploited E3 ligases in targeted protein degradation, CRBN and VHL. The distinctive contribution of this study lies in the integration of systematically optimized and validated protocols, short incubation times, implementation in both 96- and 384-well formats, and a standardized ready-to-use format. Together, these features provide a rapid, reproducible, cost-effective, and broadly accessible platform for routine affinity profiling and medium- to high-throughput screening. By enabling the early characterization of PROTACs and molecular glues, these assays complement cellular degradation studies and support a more rational, binding-first workflow for degrader discovery and optimization.

## 2. Results

### 2.1. CRBN Competition Binding Assay Development

#### 2.1.1. Design and Preliminary Characterization of Fluorescent CRBN Tracers

Fluorescence polarization (FP) is a homogeneous binding technique based on the difference in rotational diffusion between a free fluorescent ligand and the corresponding protein-bound complex. Upon binding to a macromolecular target, the reduced rotational mobility of the fluorophore produces an increase in polarization signal. Therefore, the development of a robust FP assay requires a fluorescent tracer that combines sufficient target affinity with an appropriate linker and fluorophore architecture, so that binding results in a measurable and reproducible change in polarization.

Based on these principles, we explored different CRBN-binding scaffolds and exit vectors, which were conjugated to the corresponding fluorophores through a panel of flexible (PEG- and protonatable alkyl-based) or conformationally constrained (spirocyclic- and heteroaromatic-based) linkers ranging from 7 to 14 atoms in length. CRBN-binding scaffolds were selected to preserve high-affinity CRBN binding while enabling fluorophore conjugation without compromising ligand recognition. Fluorophores were chosen to provide suitable photophysical properties while limiting intramolecular mobility. A set of 10 fluorescent CRBN tracers was generated using Celtarys’ proprietary technology, enabling systematic variation of the ligand scaffold, exit vector, linker architecture, and fluorophore.

**Figure 2:**
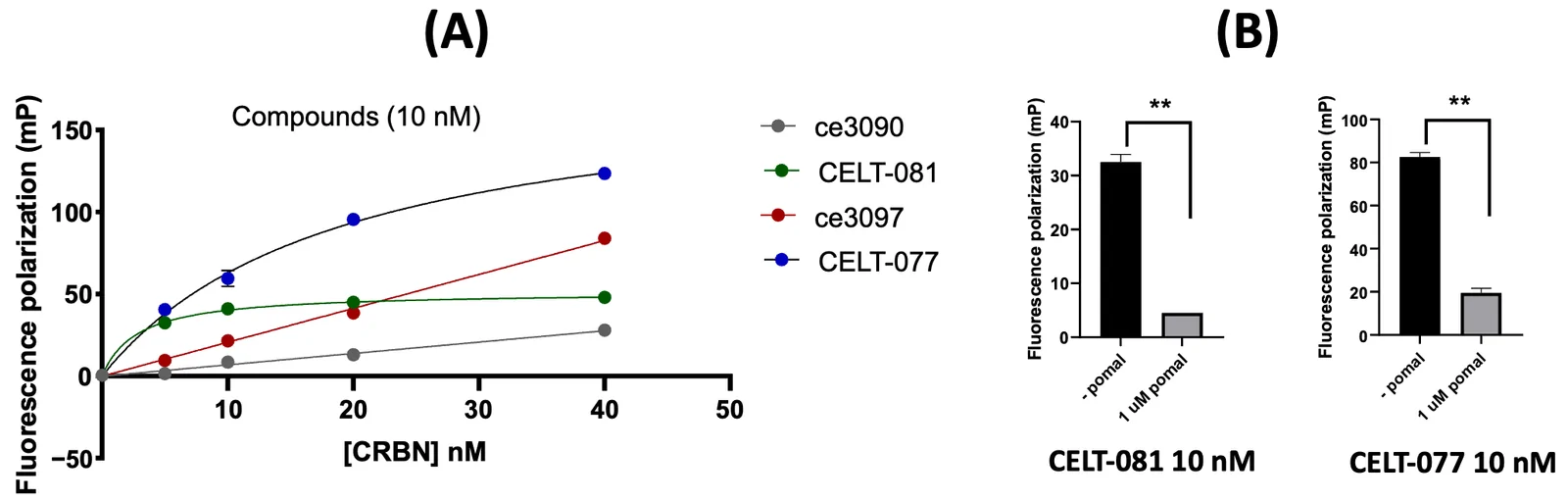
**Preliminary evaluation of representative fluorescent CRBN tracers.** (**A**) Saturation binding curves obtained by titrating increasing concentrations of recombinant CUL4A/RBX1/DDB1/CRBN complex against a fixed concentration of each fluorescent ligand, allowing assessment of tracer-dependent FP signal generation and estimation of the corresponding apparent K_d_ values. (**B**) Assay windows obtained for CELT-081 and CELT-077 in the absence or presence of 1 µM pomalidomide. Data are presented as mean ± SD of three replicates. ** *p* < 0.01, two-tailed unpaired Student’s *t*-test.

The resulting fluorescent ligands were initially evaluated by FP to determine their ability to generate a concentration-dependent polarization signal upon binding to recombinant human CRL4^CRBN^ E3 ligase complex (CUL4A/RBX1/DDB1/CRBN) and to assess whether this signal could be displaced by a reference CRBN ligand. Among the 10 tracers tested, CELT-077 and CELT-081 displayed the most favorable performance. Both tracers produced a clear increase in polarization upon titration with increasing concentrations of CRBN, indicating efficient formation of the tracer–protein complex (Figure 2A). In addition, competition with 1 µM pomalidomide produced a marked reduction in the FP signal, confirming that both tracers bind to the canonical IMiD-binding pocket and providing a suitable assay window for competitive displacement measurements (Figure 2B).

The apparent dissociation constants (*K*_d_) determined for CELT-077 and CELT-081 were 19.05 nM and 2.82 nM, respectively. These results identified both compounds as suitable candidates for further assay optimization and comparative evaluation.

#### 2.1.2. CRBN Assay Development and Characterization

To identify the most suitable fluorescent tracer for competitive binding measurements, CELT-077 and CELT-081 were directly compared using pomalidomide as a reference CRBN ligand. Tracer binding was initially characterized by protein-saturation experiments performed at a fixed fluorescent tracer concentration, a commonly used configuration for FP assay development that enables direct monitoring of the transition from free to protein-bound tracer. Based on the apparent dissociation constants obtained from these experiments, tracer concentrations were subsequently selected to provide an appropriate binding regime while maximizing assay window and sensitivity to competitive displacement. Accordingly, CELT-077 and CELT-081 were used at final concentrations of 7.5 nM and 1 nM, respectively, in the presence of 10 nM recombinant human CUL4A/RBX1/DDB1/CRBN complex. Competitive binding experiments were performed by generating concentration–response curves for pomalidomide, from which IC_50_ values were determined. The corresponding inhibition constants (Ki) were subsequently calculated using the Cheng–Prusoff equation, enabling direct comparison of the pharmacological performance of both tracers independently of their intrinsic binding affinities.

**Figure 3:**
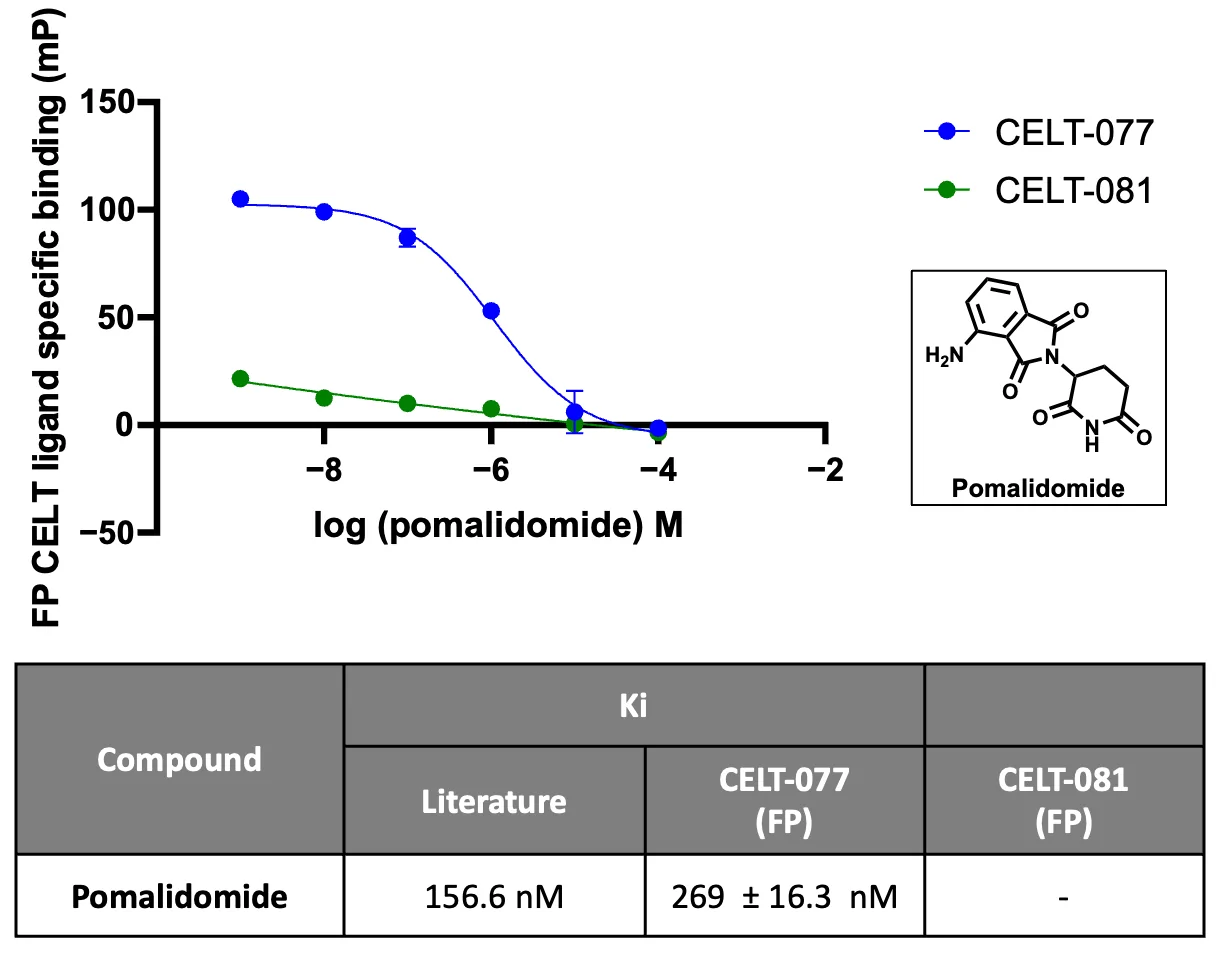
**Selection of the optimal tracer for the CRBN fluorescence polarization assay.** Competitive binding analysis of CELT-077 and CELT-081 using pomalidomide as a reference CRBN ligand. CELT-077 and CELT-081 were used at final concentrations of 7.5 nM and 1 nM, respectively, in the presence of 10 nM recombinant human CUL4A/RBX1/DDB1/CRBN complex following 10 min of incubation. Concentration–response curves were generated to determine IC_50_ values, which were converted to Ki values using the Cheng–Prusoff equation. CELT-077 accurately reproduced the reported affinity of pomalidomide and provided a substantially larger assay window (~100 mP) than CELT-081, supporting its selection as the fluorescent tracer for subsequent assay development and validation. Data are presented as mean ± SEM of three independent experiments [25].

As shown in Figure 3, the affinity determined for pomalidomide using CELT-077 was in excellent agreement with the reported literature value. By contrast, CELT-081 did not produce a sufficiently well-defined displacement curve to allow reliable IC_50_ determination and subsequent Ki calculation. Although both tracers generated measurable polarization changes upon CRBN binding, only CELT-077 combined a robust assay window with reliable pharmacological characterization, supporting its selection for subsequent assay development.

Moreover, CELT-077 provided a substantially broader assay window, with a maximum polarization shift of approximately 100 mP (Figure 3), whereas CELT-081 produced a considerably smaller change in polarization, resulting in a reduced dynamic range. Taken together, these results demonstrate that accurate affinity determination alone is not sufficient for tracer selection and that assay performance must also be considered. Accordingly, the combination of pharmacological accuracy and superior assay robustness identified CELT-077 as the optimal tracer for all subsequent assay development and validation studies.

**Figure 4:**
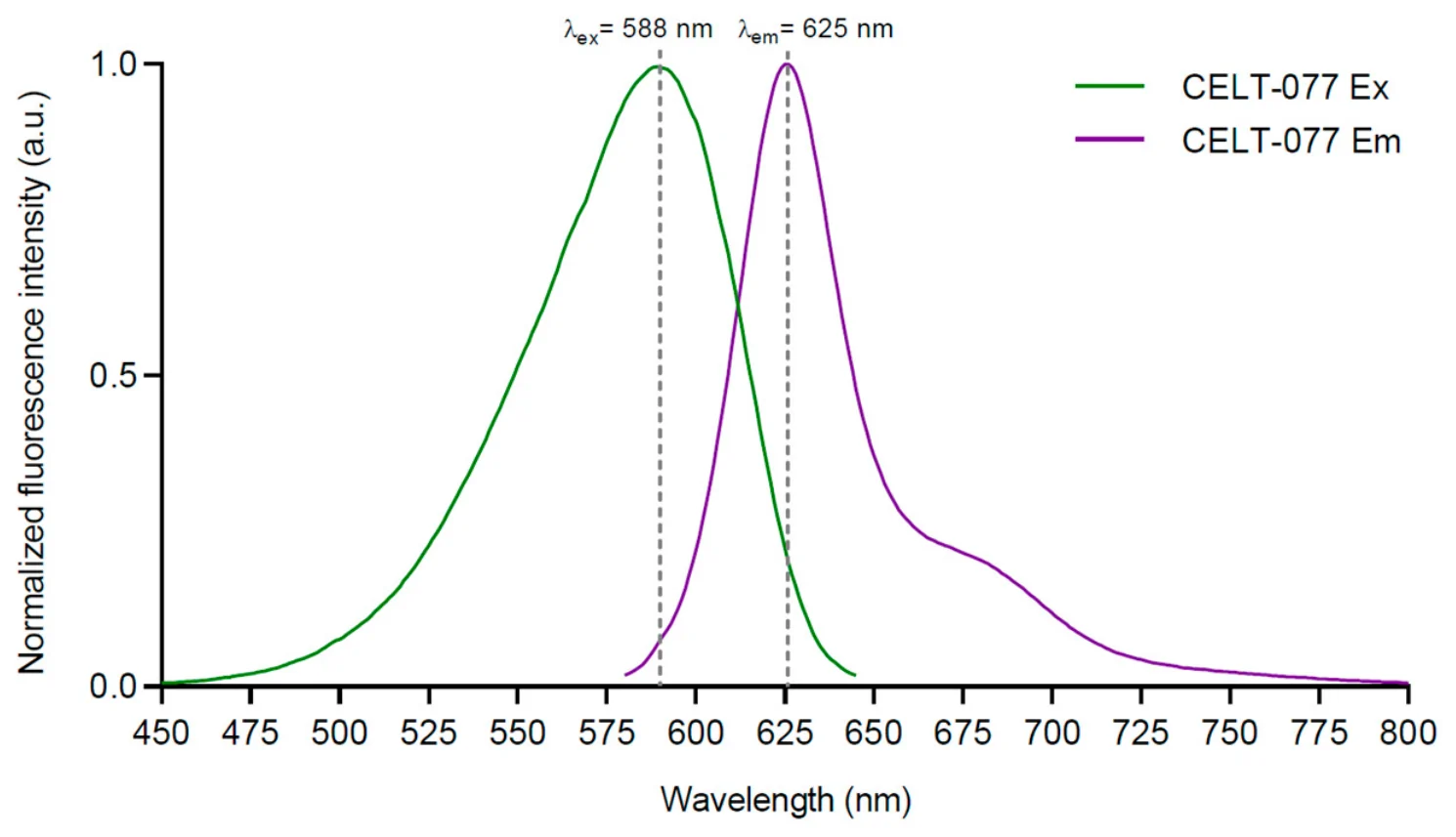
**Photophysical characterization of CELT-077.** Normalized excitation (Ex) and emission (Em) fluorescence spectra of CELT-077 in water. The excitation and emission maxima wavelengths were determined to be 588 and 625 nm, respectively.

Before proceeding with further validation, photophysical properties of CELT-077 were determined to assess its suitability for routine fluorescence polarization measurements. The tracer exhibited a maximum excitation wavelength (λmax,ex) of 588 nm and a maximum emission wavelength (λmax,em) of 625 nm (Figure 4). These properties are well suited for efficient fluorescence detection and support the use of CELT-077 as a sensitive tracer for FP-based competitive binding assays.

**Figure 5:**
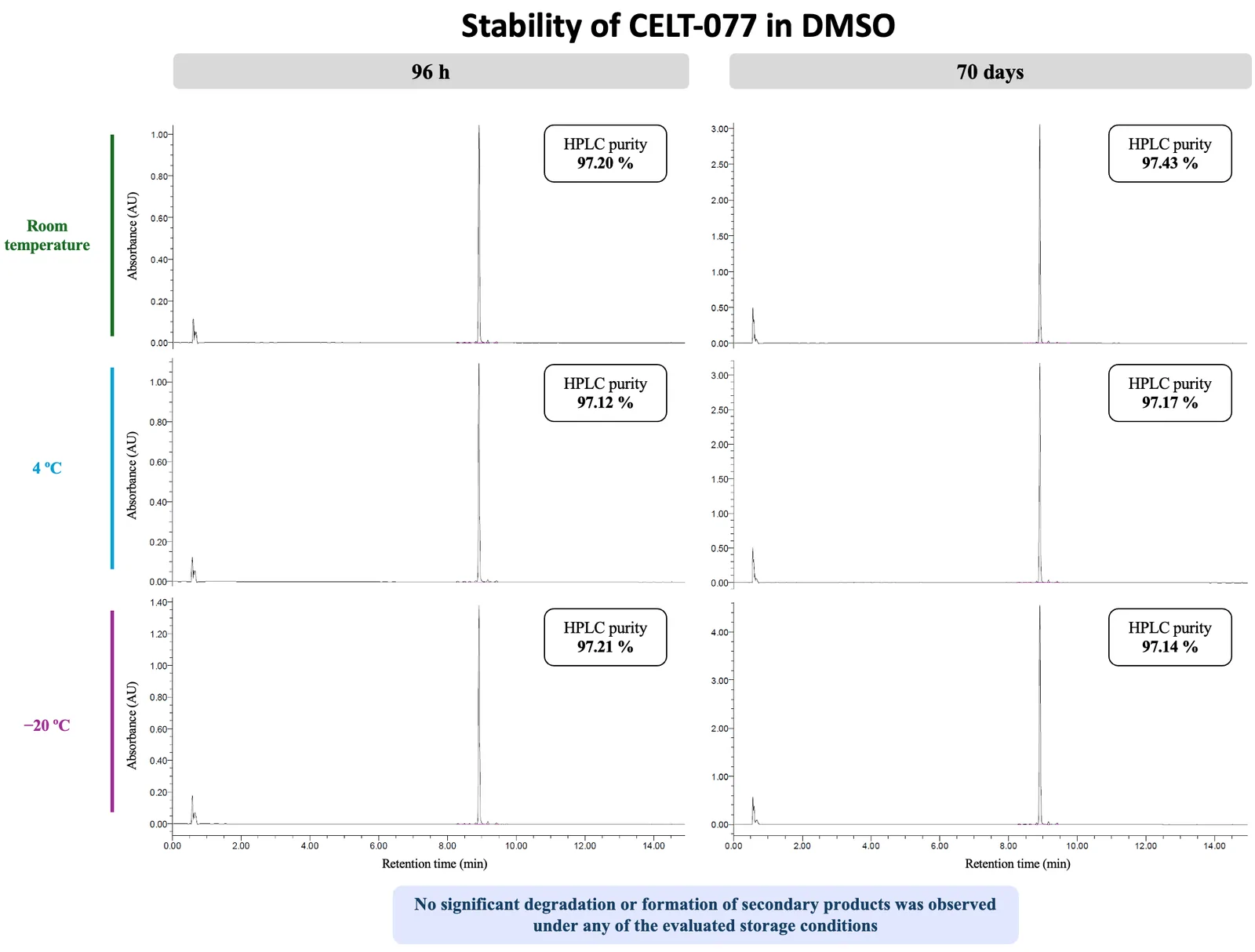
**Chemical stability of CELT-077.** HPLC chromatograms of CELT-077 dissolved in DMSO and stored at room temperature, 4 °C, or −20 °C. Samples were analyzed after 96 h and 70 days. No detectable degradation or formation of secondary products was observed under any of the storage conditions, demonstrating the excellent chemical stability of the tracer.

The chemical stability of CELT-077 was subsequently evaluated by HPLC analysis using DMSO stock solutions stored at room temperature, 4 °C and −20 °C. Chromatographic profiles recorded after 96 h and 70 days revealed no detectable degradation or formation of secondary products under any of the storage conditions examined (Figure 5). Although the tracer remained chemically stable throughout the study, storage at −20 °C was selected for routine use to ensure long-term stability of stock solutions during assay implementation.

**Figure 6:**
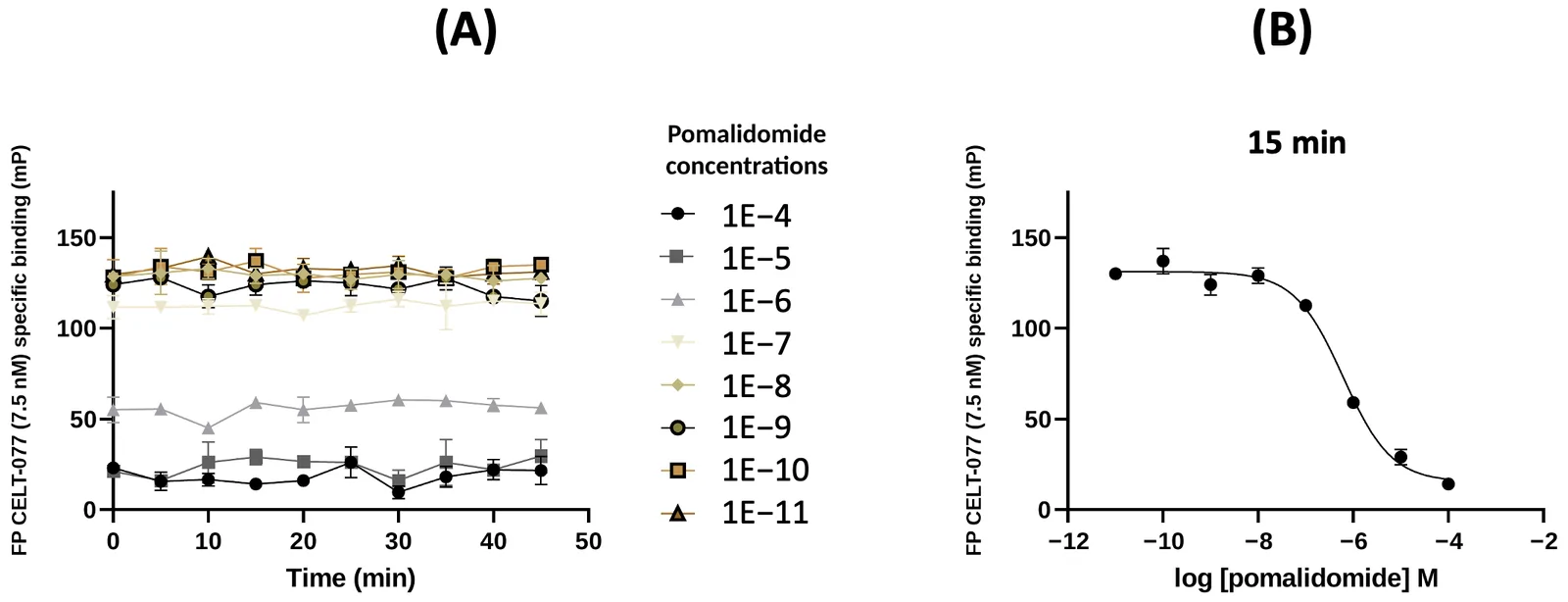
**Optimization of incubation time for the competitive fluorescence polarization assay.** (**A**) Time-course analysis of CELT-077 displacement by increasing concentrations of pomalidomide in the presence of the recombinant human CUL4A/RBX1/DDB1/CRBN complex. (**B**) Concentration-dependent displacement of CELT-077 by pomalidomide after 15 min of incubation. Data are presented as mean ± SD of duplicate wells from a representative experiment.

To establish a rapid and reproducible competitive fluorescence polarization assay, the effect of incubation time on the displacement of CELT-077 by pomalidomide was evaluated. The fluorescence polarization signal was monitored for 45 min in the presence of increasing concentrations of pomalidomide and the recombinant CUL4A/RBX1/DDB1/CRBN complex (Figure 6A). Polarization values remained essentially stable throughout the evaluated time course at all pomalidomide concentrations, indicating that binding equilibrium was reached rapidly and maintained under the assay conditions. Although the system appeared to be equilibrated within the first 10 min, an incubation time of 15 min was selected as the standard condition to provide an additional equilibration margin and improve consistency during routine assay handling, without substantially increasing the overall assay duration. At this time point, pomalidomide produced a clear concentration-dependent decrease in the specific binding signal (Figure 6B), confirming that 15 min was suitable for the subsequent competitive binding experiments.

#### 2.1.3. Validation of the CRBN Fluorescence Polarization Assay for Affinity Determination of Representative Molecular Glues and PROTACs

Following assay development and optimization, the performance of the CRBN fluorescence polarization assay was evaluated using a representative panel of CRBN-recruiting molecular glues and PROTACs spanning a broad range of reported binding affinities. Using the same workflow established for pomalidomide, concentration–response curves were generated for each compound, and the resulting IC_50_ values were converted into Ki values.

Molecular glues were investigated first since an accurate determination of the binary affinity of molecular glues for CRBN represents a fundamental step in their characterization.

**Figure 7:**
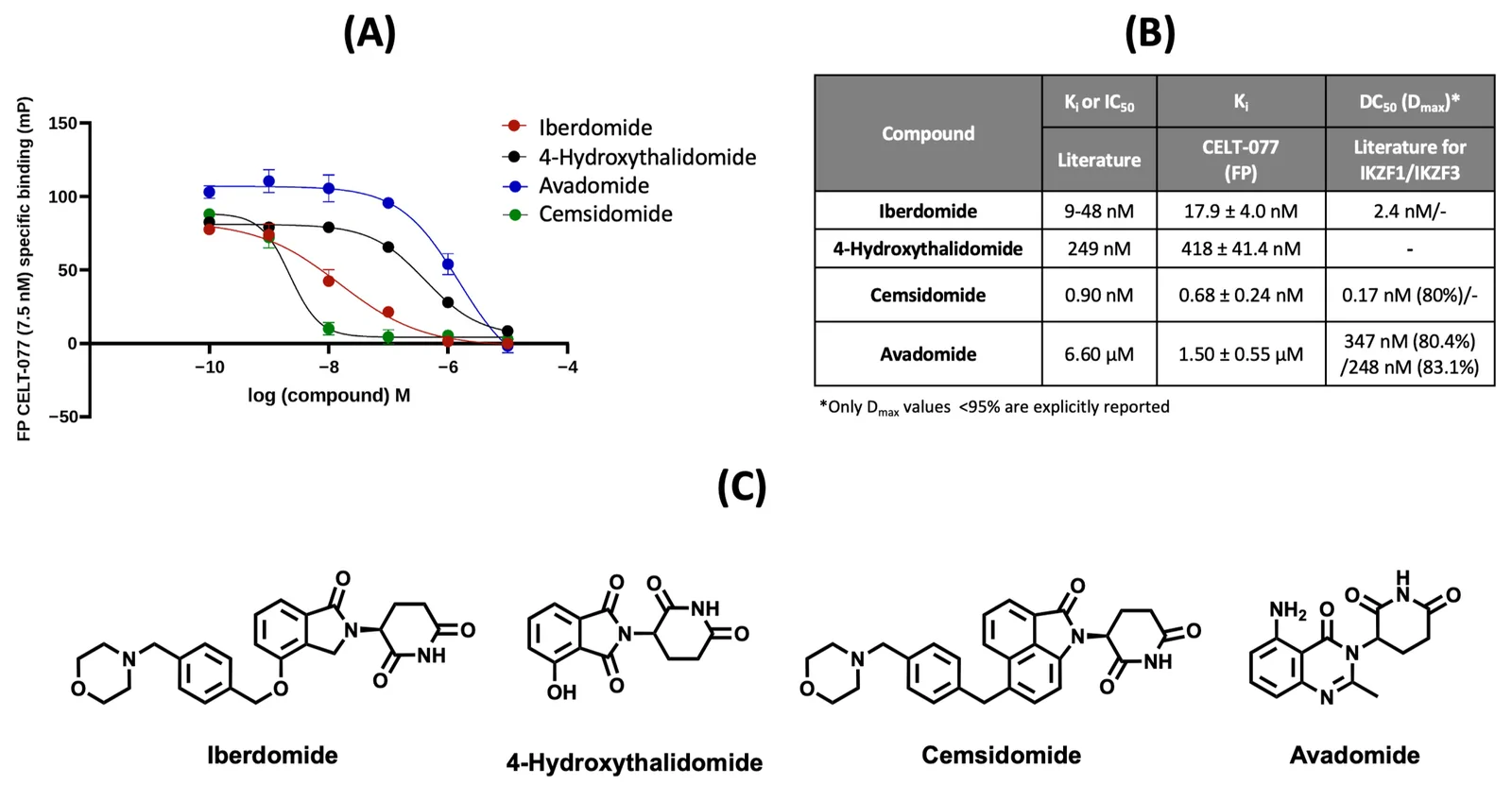
**Validation of the CRBN fluorescence polarization assay using representative molecular glues.** (**A**) Competitive fluorescence polarization binding curves obtained for the classical IMiD 4-hydroxythalidomide and the next-generation CELMoDs avadomide, iberdomide and cemsidomide. Concentration–response curves were generated using the optimized competitive FP assay. (**B**) Ki values were calculated from the corresponding IC_50_ values using the Cheng–Prusoff equation. The experimentally determined affinities were compared with previously reported literature affinities and corresponding cellular degradation potency (DC50). Data are presented as mean ± SEM (n = 4 independent experiments, each performed in duplicate). (**C**) Chemical structures of the molecular glues tested [24,25,26,27,28,29].

A representative panel comprising the classical IMiD 4-hydroxythalidomide together with the next-generation cereblon E3 ligase modulators (CELMoDs) avadomide, iberdomide and cemsidomide was selected to evaluate the ability of the assay to accurately determine binary CRBN binding affinities. As shown in Figure 7 and Table 1, all compounds generated well-defined sigmoidal concentration–response curves, allowing reliable determination of their corresponding Ki values. The assay correctly reproduced the expected affinity ranking across the series, with avadomide displaying the lowest affinity (Ki = 1.5 µM), followed by 4-hydroxythalidomide (Ki = 418 nM) and iberdomide (Ki = 17.9 nM), whereas cemsidomide exhibited the highest affinity for CRBN (Ki = 0.68 nM). Notably, the experimentally determined Ki values for all four molecular glues were in excellent agreement with previously reported data, with deviations of less than 5-fold from the corresponding literature values. The close agreement between experimentally determined and literature values demonstrates the robustness and accuracy of the assay for quantitative affinity determination of CRBN molecular glues. For comparison, the experimentally determined Ki values are presented alongside the corresponding reported biological activities in Figure 7, providing a direct framework to relate binary CRBN affinity to the pharmacological performance of representative molecular glues.

**Figure 8:**
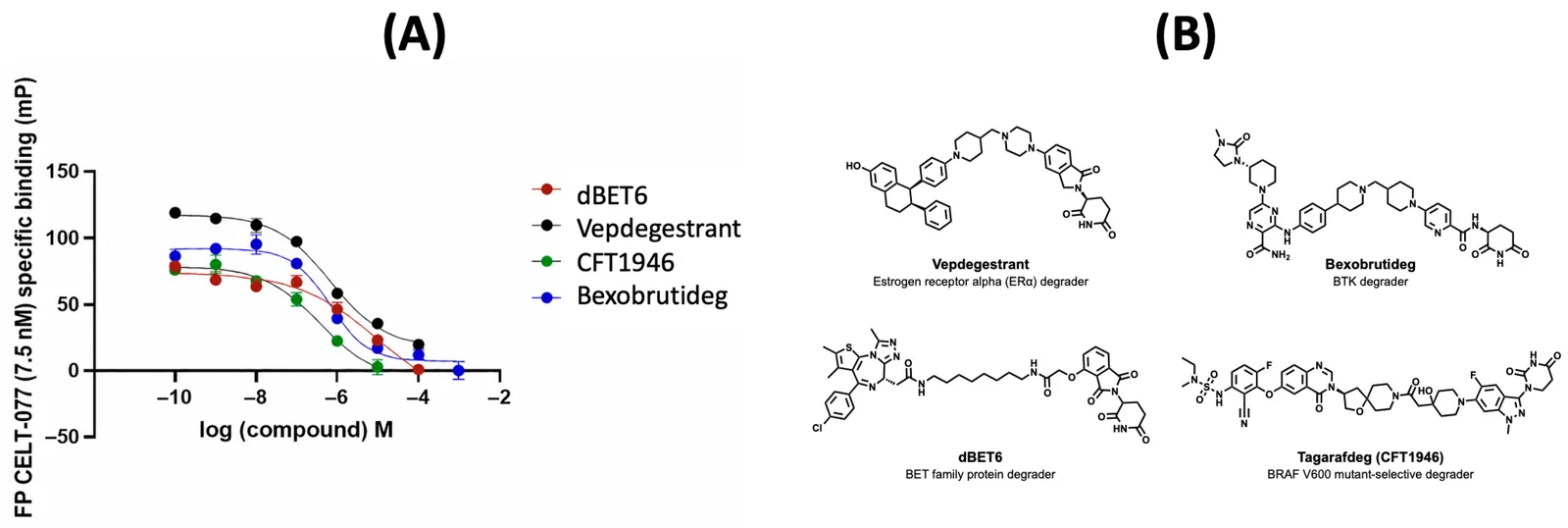
**Validation of the CRBN fluorescence polarization assay using representative PROTACs and assay miniaturization.** (**A**) Competitive fluorescence polarization concentration–response curves obtained for the selected CRBN-recruiting PROTACs using the optimized assay in the 96-well plate format. (**B**) Chemical structures of the CRBN-recruiting PROTACs included in the validation panel. Data are presented as mean ± SEM (*n* = 3 independent experiments, each performed in duplicate).

Following validation with molecular glues, the applicability of the assay was extended to representative CRBN-recruiting PROTACs. The clinical success of orally bioavailable CRBN-recruiting degraders has established CRBN as the most extensively exploited E3 ligase for PROTAC development, highlighting the need for robust methods to quantify binary CRBN affinity. A panel of structurally diverse reported PROTACs was therefore selected for evaluation (Figure 8). The selected degraders were chosen to maximize structural diversity, encompassing different POI ligands, linker architectures, and CRBN-recruiting moieties. In addition to classical pomalidomide-based recruiters, the panel included more recent CRBN ligands specifically designed to improve chemical stability and remove stereocenters, such as the phenyl-dihydrouracil recruiter incorporated in CFT1946. Despite the substantial structural differences between molecular glues and PROTACs, using the optimized competitive fluorescence polarization assay protocol established for molecular glues, all PROTACs generated well-defined sigmoidal concentration–response curves, allowing reliable determination of their corresponding Ki values. For all degraders with previously reported CRBN affinity data, the experimentally determined Ki values were within a 3-fold difference from the corresponding literature values, demonstrating that the assay accurately quantifies binary CRBN binding independently of degrader size, linker composition, or recruiter scaffold (Figure 8 and Table 1). In addition, the assay enabled determination of the binary CRBN affinity of Bexobrutideg, for which no experimental CRBN binding affinity has previously been reported, thereby extending the available pharmacological characterization of this clinically relevant degrader. Likewise, the experimentally determined Ki values are presented together with the corresponding reported cellular degradation potencies (DC50) (Table 1), enabling direct comparison between binary CRBN affinity and degradation efficiency across representative degraders.

**Table 1:** Experimentally determined CRBN binding affinities of representative CRBN-recruiting PROTACs measured using the optimized fluorescence polarization assay and comparison with corresponding reported degradation potencies.

Compound	K_i_ or IC_50_	K_i_		DC_50_ (D_max_) ^1^
	Literature	CELT-077 (FP) 96-Well Format	CELT-077 (FP) 384-Well Format	Literature
Pomalidomide	156 nM [25]	230 ± 10 nM	300 ± 30 nM	375 nM (IKZF1)/ 807 nM (IKZF3) [30]
Vepdegestrant	123 nM [31]	87 ± 2 nM	86 ± 9 nM	0.90 nM [2]
Bexobrutideg	-	600 ± 100 nM	230 ± 9 nM	0.34 nM [32]
Tagarafdeg (CFT1946)	400 nM [33]	300 ± 30 nM	460 ± 70 nM	14.0 nM (75%) [34]
dBET6	156 nM [31]	380 ± 10 nM	378 ± 7 nM	10.0 nM [35]

^1^ Only D_max_ values < 95% are explicitly reported. For PROTACs, Ki values obtained in both the 96- and 384-well plate formats are compared with previously reported literature affinities and cellular degradation potencies (DC_50_). Pomalidomide was included in every experiment as an internal assay control. Data are presented as mean ± SEM (*n* = 3 independent experiments, each performed in duplicate). Standard errors associated with the nonlinear regression-derived IC_50_ values were propagated to the corresponding Ki values. Literature cellular degradation potencies (DC_50_) are provided for contextual comparison only [2,25,30,31,32,33,34,35].

#### 2.1.4. Miniaturization of the CRBN Fluorescence Polarization Assay

The robustness and reproducibility of the optimized assay enabled its direct miniaturization to a 384-well plate format without modification of the experimental workflow. Furthermore, the 384-well format matches the standard configuration of most modern fluorescence plate readers, facilitating straightforward implementation of the assay in both academic laboratories and industrial high-throughput screening platforms. Miniaturization represents an important step toward high-throughput screening applications, as it substantially increases assay throughput while reducing reagent consumption and overall experimental costs. In particular, the lower reaction volume markedly decreases the amounts of fluorescent tracer, recombinant CRL4^CRBN^ complex, and assay buffer required per determination, allowing approximately four times more compounds to be evaluated using approximately the same total amount of reagents. Importantly, assay miniaturization did not compromise analytical performance. The Ki values obtained in the 384-well format were in excellent agreement with those determined in the 96-well format and remained within a 3-fold difference from the corresponding literature values whenever reference affinity data were available (Table 1). Likewise, the affinity determined for Bexobrutideg showed good agreement between the 96- and 384-well formats, further supporting the robustness of the miniaturized assay.

### 2.2. VHL Competition Binding Assay Development

**Figure 9:**
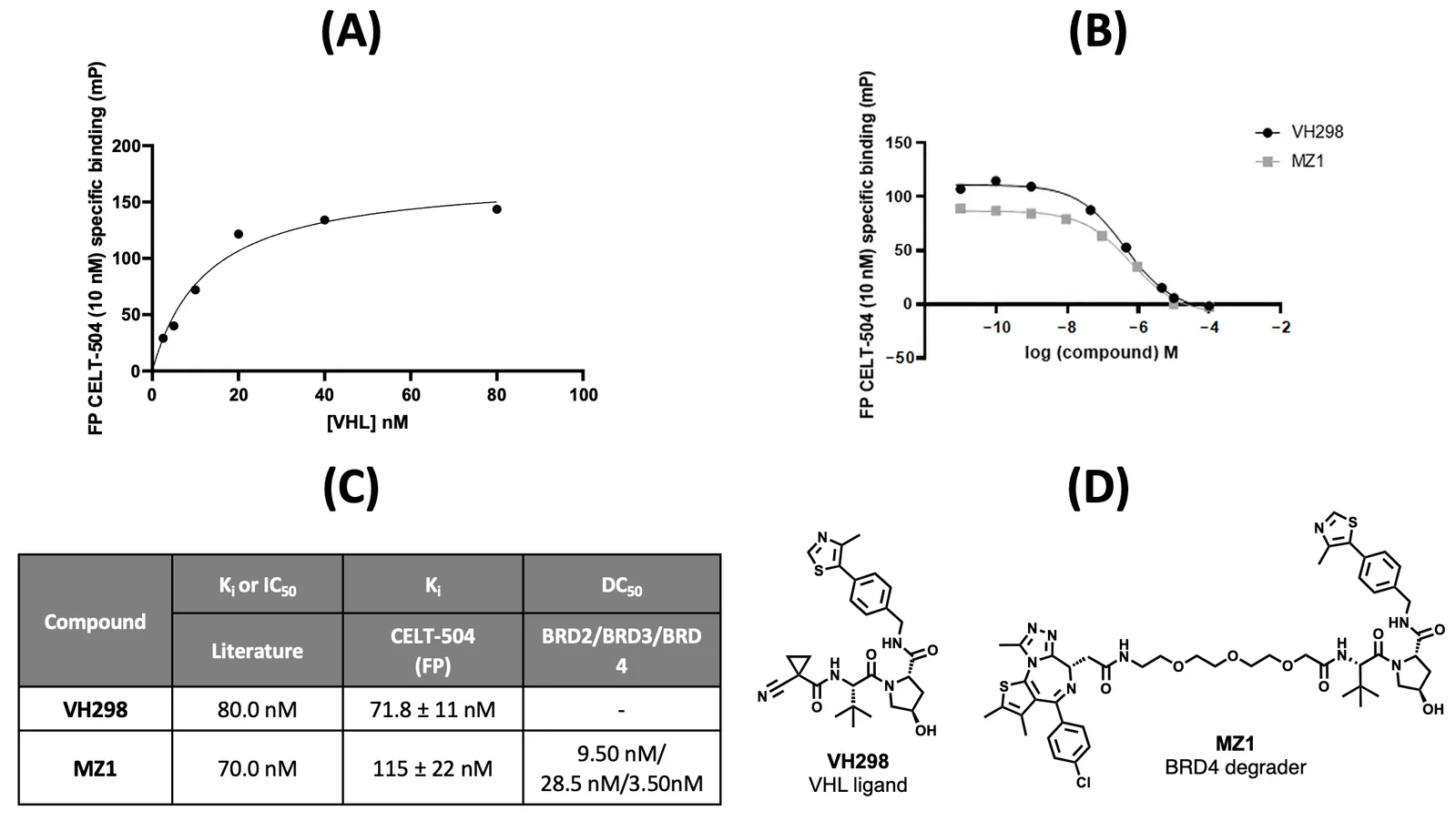
**Validation of the VHL fluorescence polarization assay.** (**A**) Saturation curve of CELT-504. (**B**) Competitive fluorescence polarization concentration–response curves obtained for the VHL ligand VH298 and the VHL-recruiting BET degrader MZ1 using the optimized assay in 384-well format. (**C**) Comparison of the experimentally determined Ki values obtained in the 96- and 384-well plate formats with previously reported literature affinities. For MZ1, the reported cellular degradation potency (DC_50_) is also shown to facilitate comparison between binary VHL affinity and degradation activity. Data are presented as mean ± SEM (*n* = 3 independent experiments, each performed in duplicate). (**D**) Molecular structures of VH298 and MZ1 [36,37].

A similar strategy was applied to develop a fluorescence polarization assay for the von Hippel–Lindau (VHL) E3 ligase. Using Celtarys’ proprietary design platform, a set of 8 fluorescent VHL ligands was generated by systematically varying the exit vector within the 4-hydroxyproline-based scaffold, linker nature and architecture, and fluorophore. Following experimental evaluation, CELT-504 was identified as the optimal fluorescent tracer based on its binding properties, with a K_d_ of 12.6 nM (Figure 9A), and assay performance, and was therefore selected for subsequent assay development.

**Figure 10:**
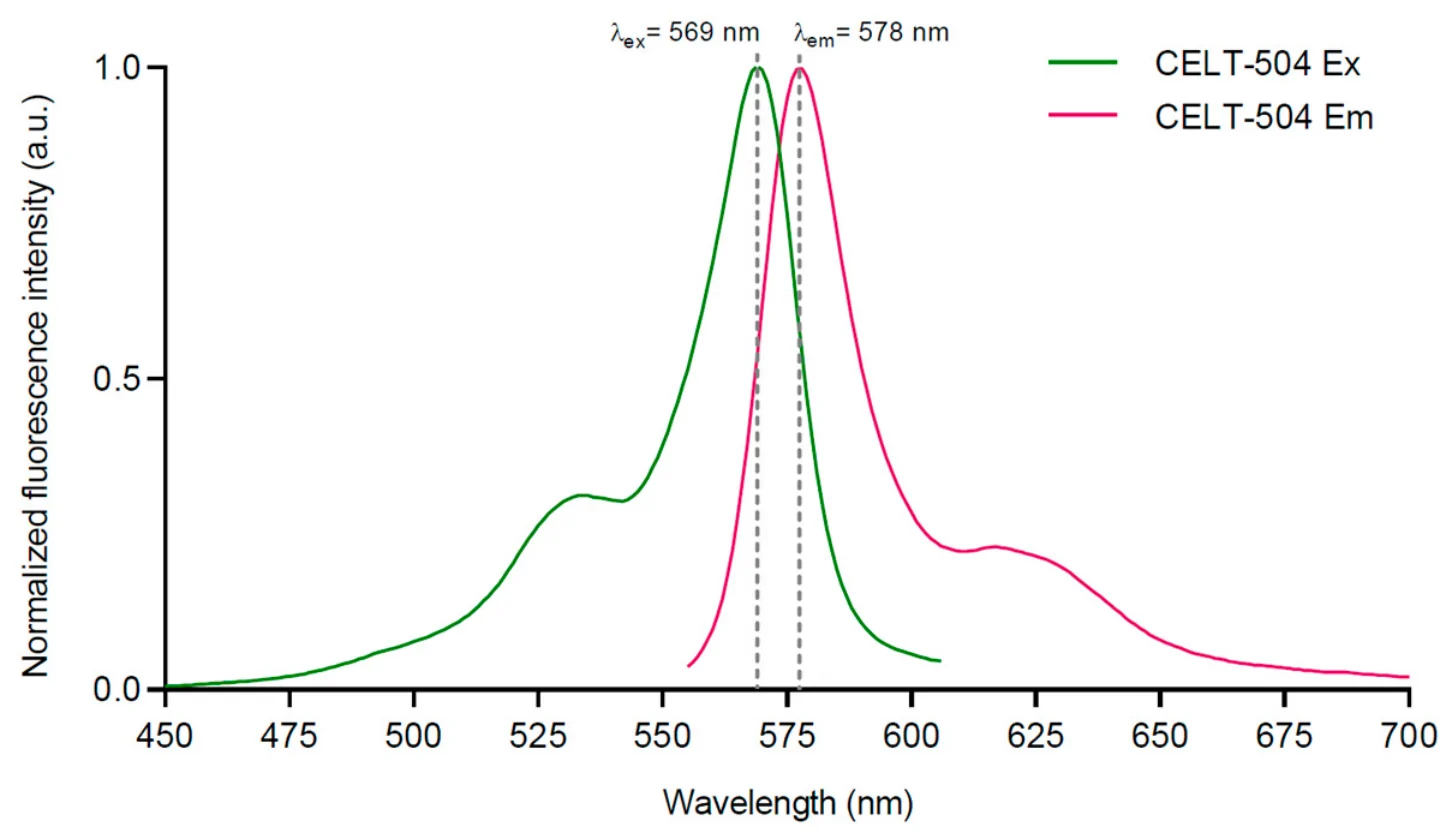
**Photophysical characterization of CELT-504.** Normalized excitation (Ex) and emission (Em) fluorescence spectra of CELT-504 in water. The excitation and emission maxima wavelengths were determined to be 569 and 578 nm, respectively.

The photophysical properties of CELT-504 were subsequently characterized, revealing excitation and emission maxima at 569 nm and 578 nm, respectively (Figure 10).

The validation strategy for the CRBN binding assay prioritized clinically advanced PROTACs and well-characterized molecular glues with reliable pharmacological data. However, only a limited number of VHL-recruiting PROTACs have reached clinical evaluation, and well-characterized VHL molecular glues remain extremely scarce. Consequently, the initial VHL validation was performed using the canonical VHL ligand VH298 and BET degrader MZ1, one of the most extensively characterized VHL-recruiting PROTACs. Both compounds generated well-defined concentration–response curves in the competitive fluorescence polarization assay (Figure 9B), allowing reliable determination of their corresponding Ki values. The experimentally determined affinities obtained in both the 96- and 384-well plate formats were in good agreement with previously reported literature values (Figure 9C). Comparison of the experimentally determined VHL affinity with the previously reported BRD degradation potency of MZ1 further illustrates how binary binding data can be interpreted alongside degradation outcomes, supporting the use of early E3 ligase affinity measurements to guide degrader optimization (Figure 9C).

## 3. Discussion

The results presented here highlight the value of binary E3 ligase affinity as an orthogonal parameter for degrader profiling during early drug discovery. Comparison of the experimentally determined binary E3 ligase affinities with the corresponding reported degradation potency suggests that binary affinity measurements provide valuable mechanistic information that cannot be obtained from cellular degradation assays alone. As an illustrative example, inspection of the representative CRBN degraders included in Table 1 indicates that compounds displaying potent cellular degradation generally exhibit binary CRBN affinities within a submicromolar affinity window (approximately ≤ 500 nM). Although this empirical observation should not be interpreted as a universal predictive threshold, it illustrates how binary affinity measurements may help define practical affinity windows for medicinal chemistry optimization, as both weak and excessively strong binary interactions may contribute to poor degradation or concentration-dependent hook-effect behavior. Because cellular degradation efficiency depends on multiple additional factors—including target affinity, ternary complex cooperativity, linker composition, intracellular exposure, and ubiquitination efficiency—binary E3 ligase affinity alone cannot predict degrader potency. Nevertheless, rapid affinity determination represents a rational first-pass filter, supporting a binding-first, rather than a degradation-first, workflow. Beyond compound profiling, these fluorescence polarization assays provide practical and scalable platforms for the discovery, characterization, and optimization of novel E3 ligase recruiters. Although CRBN recruiters are also largely derived from a single privileged chemotype, their favorable drug-like properties have enabled the successful development of numerous orally bioavailable degraders, with the majority of degraders currently in clinical development relying on CRBN recruitment. In contrast, VHL recruiters remain predominantly based on hydroxyproline-derived ligands, whose physicochemical properties continue to pose significant challenges for medicinal chemistry optimization and the development of orally bioavailable degraders. Likewise, molecular glues have become one of the fastest-growing areas of targeted protein degradation. Whereas CRBN has emerged as the predominant E3 ligase for molecular glue discovery, this modality remains largely unexplored for VHL. The recent discovery of the first VHL-directed molecular glue inducing degradation of cysteine dioxygenase 1 (CDO1) further highlights the opportunity to expand this emerging therapeutic strategy [38]. In this context, the fluorescence polarization assays described here may facilitate both the discovery of alternative CRBN and VHL recruiter chemotypes and the identification and characterization of future molecular glues targeting these E3 ligases. The fluorescence polarization assays developed in this work provide robust, reproducible, and miniaturized platforms for quantitative determination of CRBN and VHL binary affinity. Their successful implementation in both 96- and 384-well formats demonstrates the scalability of the methodology and enabled the establishment of a standardized, ready-to-use assay format that facilitates routine affinity profiling and straightforward adoption across different laboratories. Although both assays underwent tracer selection, optimization, and miniaturization, the VHL assay was validated using a smaller and less chemically diverse compound panel than the CRBN assay, due to the lack of well-characterized degraders and glues in clinical trials. Future studies will expand the panel to include new chemical classes, clinical-stage VHL PROTACs, novel VHL recruiters, and emerging VHL molecular glues as suitable compounds and pharmacological data become available. Beyond binary affinity determination, the fluorescence polarization platform presented here also provides a versatile foundation for future assay development. Ongoing work in our laboratory is focused on extending this platform to fluorescence polarization-based assays for ternary complex cooperativity and target engagement, ultimately establishing an integrated screening toolbox to support targeted protein degrader discovery and optimization. In parallel, we are expanding this assay platform to additional E3 ligases, including DCAF family members, with the aim of broadening its applicability for the discovery and characterization of next-generation E3 ligase recruiters.

## 4. Materials and Methods

**Excitation and emission spectra**. CELT-077 (10 μg) and CELT-504 (5 μg) were individually dissolved in 200 μL of DMSO. Each solution was transferred to a 10 mm path-length quartz cuvette (Starna Scientific, Ilford, UK) and diluted to a final volume of 3.0 mL with Milli-Q water, resulting in final probe concentrations of 3.0 μM and 1.5 μM, respectively. Fluorescence excitation and emission spectra were recorded at room temperature using an Edinburgh Instruments FS5 spectrofluorometer (Edinburgh Instruments, Livingston, UK) equipped with a xenon lamp. Excitation and emission scans were acquired with a scan step of 1 nm and a dwell time of 0.30 s. For CELT-077, excitation and emission spectra were recorded over wavelength ranges of 440–645 nm and 580–800 nm, respectively, whereas for CELT-504 the corresponding ranges were 440–620 nm and 555–700 nm. Spectra were exported, normalized to their maximum fluorescence intensity, and plotted using GraphPad Prism version 9.3.1 (GraphPad Software, San Diego, CA, USA).

**Stability studies.** CELT-077 (50 μg) was dissolved in DMSO to obtain a 200 μM stock solution. An aliquot of the freshly prepared solution was analyzed immediately and used as the reference sample (t0). The remaining solution was divided into three equal aliquots and stored at room temperature, 4 °C, or −20 °C. Samples were analyzed after 96 h and 70 days of storage. Prior to analysis, aliquots were diluted with Milli-Q water and transferred to HPLC vials fitted with glass inserts. Chromatographic analyses were performed using a Waters ACQUITY Arc system coupled to a Waters ACQUITY QDa single quadrupole mass detector equipped with an electrospray ionization (ESI) source operating in positive-ion mode. Separation was achieved on an XBridge Premier BEH C18 column (2.5 μm, 2.1 × 100 mm) maintained at 40 °C using a flow rate of 0.4 mL min^−1^. The mobile phase consisted of water containing 0.1% formic acid (solvent A) and acetonitrile (solvent B). A linear gradient from 5 to 100% solvent B was applied over 15 min, followed by a 3 min column re-equilibration under the initial mobile-phase conditions. The injection volume was 10 μL. Chromatograms were extracted simultaneously at 300 and 580 nm. Stability was assessed by comparing the chromatographic purity, retention time, and the appearance of degradation products with those of the freshly prepared reference sample.

**Reagents and recombinant proteins.** The fluorescent tracers CELT-077, CELT-081, and CELT-504 were supplied by Celtarys Research (Santiago de Compostela, Spain). All fluorescence polarization measurements were performed in a saline assay buffer at pH 7.

Recombinant human CUL4A/RBX1/DDB1/CRBN complex, comprising CUL4A, RBX1, DDB1, and CRBN (R&D Systems, a Bio-Techne brand, Minneapolis, MN, USA; catalog no. E3-650), and recombinant human ELOB/ELOC/VHL complex (R&D Systems, a Bio-Techne brand; catalog no. E3-600) were used as the target proteins. Protein aliquots were thawed on ice immediately before use, maintained on ice during reagent preparation, and protected from repeated freeze–thaw cycles. Fluorescent tracers were protected from light throughout handling. Test compounds were prepared as stock solutions in dimethyl sulfoxide (DMSO) and diluted to the required working concentrations. The CRBN compound panel comprised pomalidomide, 4-hydroxythalidomide, iberdomide, avadomide, cemsidomide, vepdegestrant, bexobrutideg, CFT1946 (Tagarafdeg), and dBET6, as applicable to each experiment. VH298 and MZ1 were evaluated in the VHL assay. Matched vehicle controls were included on every plate, and the solvent contribution was maintained constant across each dose–response series.

**Microplate formats and general assay setup.** FP assays were conducted in black, nonbinding-surface microplates. The 96-well format used Corning 96-well black polystyrene NBS microplates (catalog no. 3560) with a final assay volume of 100 µL per well. The miniaturized 384-well format used Corning 384-well low-volume black round-bottom polystyrene NBS microplates (catalog no. 4514) with a final assay volume of 20 µL per well. The 384-well format retained the same final reagent concentrations and the same 2:1:1 volumetric ratio of protein solution, compound/vehicle solution, and tracer solution used in the 96-well format. Accordingly, 96-well assays were assembled by sequentially adding 50 µL of protein solution or buffer, 25 µL of a 4× compound or vehicle solution, and 25 µL of tracer solution or tracer vehicle. For 384-well assays, the corresponding volumes were 10, 5, and 5 µL, respectively. All concentrations reported below refer to the final concentration in the assay well. Assay reagents were equilibrated to room temperature before plate assembly, while protein and tracer solutions were kept on ice until addition.

**CRBN Fluorescence polarization assay.**

**Tracer binding characterization.** Binding of fluorescent CRBN tracers was characterized by fluorescence polarization using a fixed tracer concentration and increasing concentrations of recombinant CUL4A/RBX1/DDB1/CRBN complex. The resulting protein-saturation curves were used to estimate apparent dissociation constant (K_d_) values and to characterize the tracer-dependent FP response. These binding data were subsequently used, together with assay window and competitive displacement performance, to guide selection of the tracer concentrations employed in the competition assays.

**Tracer selection.** CELT-077 and CELT-081 were initially compared as CRBN tracers using 10 nM recombinant CUL4A/RBX1/DDB1/CRBN complex and 10 nM fluorescent tracer. Pomalidomide [25] was used as the reference competitor, and concentration–response curves were generated under otherwise identical assay conditions. CELT-077 was selected for subsequent compound profiling on the basis of its pharmacological performance and assay window.

**Compound displacement measurements.**

**CRBN Fluorescence polarization assay.** For routine CRBN displacement experiments, recombinant CUL4A/RBX1/DDB1/CRBN complex and CELT-077 were used at final concentrations of 10 nM and 7.5 nM, respectively. Pomalidomide [25] served as the reference competitor. Test compounds were analyzed as serial concentration–response curves using solvent-matched 4× working solutions. After addition of protein and compound solutions, plates were mixed gently for 15 min without generating bubbles. CELT-077 was then added, and plates were covered, protected from light, and mixed for a further 15 min before FP measurement. FP was measured using an Infinite M1000 multimode microplate reader (Tecan, Männedorf, Switzerland) in parallel and perpendicular detection channels, with fixed gain, G-factor, and optical settings across each plate. For CELT-077, excitation was set at approximately 590 nm and emission was collected at 616 nm, according to the optical configuration used for each experiment. The wavelength pair providing the largest and most reproducible difference between the free-tracer and protein-bound controls was selected before acquisition of the full plate.

**VHL fluorescence polarization displacement assay.** VHL binding was measured using recombinant ELOB/ELOC/VHL complex and the fluorescent tracer CELT-504 at final concentrations of 20 and 10 nM, respectively. VH298 [36] was used as the reference VHL ligand, and MZ1 was included as a VHL-recruiting degrader. Compound concentration–response series were prepared as 4× working solutions and evaluated using the same plate-assembly scheme described above. After combining protein and compound solutions, plates were mixed gently for 15 min. CELT-504 was subsequently added, and the plates were covered, protected from light, and mixed for an additional 15 min before FP acquisition. FP was recorded in top-read mode using the Infinite M1000 multimode microplate reader (Tecan), with parallel and perpendicular detection channels and a fixed gain setting throughout the plate. CELT-504 was measured using excitation and emission wavelengths of 530 and 570 nm, respectively.

**Data processing and affinity calculations.** Raw polarization values were expressed in millipolarization units (mP). The specific binding signal for each well was normalized to the FPmin and FPmax controls according to:

(1) % specific binding = 100 × (mPsample − mean mPFPmin)/(mean mPFPmax − mean mPFPmin)

Normalized binding was plotted against the logarithm of compound concentration and analyzed by nonlinear regression using the four-parameter logistic model “log(inhibitor) vs. response–variable slope” in GraphPad Prism:

(2) Y = Bottom + (Top − Bottom)/(1 + 10^((LogIC_50_ − X) × HillSlope))

where Y represents the normalized binding response; X is the base-10 logarithm of the inhibitor concentration; Bottom and Top are the lower and upper asymptotes of the fitted curve, respectively; LogIC_50_ is the logarithm of the concentration producing a response halfway between Top and Bottom; and HillSlope describes the steepness of the concentration–response curve. IC_50_ values and their associated standard errors were obtained from the nonlinear regression analysis.

Inhibition constants (Ki) were calculated from the measured IC_50_ values using the Cheng–Prusoff relationship:

(3) Ki = IC_50_/(1 + [L]/Kd)

where [L] is the final fluorescent-tracer concentration and Kd is the dissociation constant of the tracer under the assay conditions. For CELT-504, a Kd of 2.3 nM was used; therefore, with 10 nM CELT-504, Ki = IC_50_/5.35. For CELT-077, a Kd of 20 nM was used; therefore, with 7.5 nM CELT-077, Ki = IC_50_/1.375.

Each plate included a buffer-only blank, an FPmin control containing tracer in the absence of protein, an FPmax control containing protein and tracer in the absence of competitor, a protein-only control, and compound-only or vehicle controls where required to assess intrinsic fluorescence or optical interference. FPmax is defined here as the high-polarization control obtained with tracer and protein in the absence of competitor and should not be interpreted as a universal theoretical maximum or as complete tracer saturation. Absolute FP values are specific to each tracer–protein pair and depend on the rotational properties of the complex, fluorophore photophysical properties, and local fluorophore mobility. The total fluorescence intensity in the parallel and perpendicular channels was inspected alongside the polarization signal to identify potential quenching, precipitation, or compound autofluorescence. Concentrations were measured in at least duplicate wells, and each assay was independently repeated two or three times. Curve fitting and graphical analysis were performed using GraphPad Prism (GraphPad Software, Boston, MA, USA). Data points in concentration–response curves are presented as mean ± SEM of the indicated number of independent experiments, each performed with technical replicates, as specified in the corresponding figure legends. Standard errors reported for IC_50_ and Ki values correspond to the uncertainty associated with the nonlinear regression-derived parameter estimates.

## 5. Conclusions

In this work, we developed rapid and reproducible fluorescence polarization assays for the quantitative determination of binary ligand binding to CRBN and VHL. The CRBN assay was validated using molecular glues, CELMoDs, and representative PROTACs, including clinical-stage degraders, whereas the smaller VHL panel comprised VH298 and MZ1, reflecting the limited number of clinically advanced VHL-recruiting PROTACs. The experimentally determined affinities were consistent with reported values, and both assays were successfully implemented in 96- and 384-well formats. These assays provide a practical and scalable tool for implementing a binding-first workflow in degrader discovery. By enabling the early identification and prioritization of compounds that effectively bind the E3 ligase, they can help reduce the number of candidates advanced to more resource-intensive cellular degradation assays, thereby increasing the efficiency and throughput of degrader development. Rather than replacing degradation studies, binary affinity measurements complement them by providing an early and mechanistically informative criterion for compound selection.

## Data Availability

The original contributions presented in this study are included in the article. Further inquiries can be directed to the corresponding authors.

## Abbreviations

The following abbreviations are used in this manuscript:

BET	Bromodomain and Extra-Terminal
BRET	Bioluminescence Resonance Energy Transfer
CRBN	Cereblon
CRL4	Cullin-RING Ligase 4
DDB1	DNA Damage-Binding Protein 1
DC_50_	Half-Maximal Degradation Concentration
DMSO	Dimethyl Sulfoxide
E3	E3 Ubiquitin Ligase
ELOB	Elongin B
ELOC	Elongin C
ER	Estrogen Receptor
ERK	Extracellular Signal-Regulated Kinase
ESI	Electrospray Ionization
FDA	Food and Drug Administration
FP	Fluorescence Polarization
HER2	Human Epidermal Growth Factor Receptor 2
HPLC	High-Performance Liquid Chromatography
IC_50_	Half-Maximal Inhibitory Concentration
IMiDs	Immunomodulatory Imide Drugs
Kd	Equilibrium Dissociation Constant
Ki	Inhibition Constant
MGDs	Molecular Glue Degraders
mP	Millipolarization Unit
NBS	Non-Binding Surface
PEG	Polyethylene Glycol
POI	Protein of Interest
PROTACs	PROteolysis-TArgeting Chimeras
QDa	Quadrupole Detector Array
RBX1	RING-Box Protein 1
SAR	Structure–Activity Relationship
SD	Standard Deviation
TPD	Targeted Protein Degradation
UPS	Ubiquitin–Proteasome System
VHL	von Hippel–Lindau
λmax,em	Maximum Emission Wavelength
λmax,ex	Maximum Excitation Wavelength
